# Proficiencies of military medical officers in intubating difficult airways

**DOI:** 10.1186/s12873-020-00375-2

**Published:** 2020-10-07

**Authors:** Jonathan ZM Lim, Shi Hao Chew, Benjamin ZB Chin, Raymond CH Siew

**Affiliations:** 1 Department of Anaesthesia, National University Health System, 5 Lower Kent Ridge Rd, Singapore, 119074 Singapore; 2 RS Anaesthesia & Intensive Care, 71 Ubi Road 1, #05-41, Singapore, 408732 Singapore

**Keywords:** Trauma, Airway, Manikin, Junior doctors, Laryngoscopes

## Abstract

**Background:**

This study sheds light on the proficiency of military medical officers who had received between 2 and 3 years of post-graduate training, in the handling of the difficult airway in a trauma manikin simulator using direct and video laryngoscopes.

**Method:**

One hundred thirty-three doctors from the Singapore Armed Forces Medical Officer Cadet Course were assessed using high-fidelity simulator models with standardised difficult airways (simulator with tongue-swelling and cervical collar). They used the Macintosh direct laryngoscope (DL), King Vision channelled-blade laryngoscope (KVC), King Vision non-channelled blade laryngoscope (KVNC), and the McGrath (MG) laryngoscope on the same model in a randomised sequence. The intubation success rates and time to intubation were recorded and analysed for the study.

**Results:**

The medical officers had a 71.4% intubation success rate with the DL on the difficult airway trauma simulator model and the mean time to intubation of 40.1 s. With the KVC, the success rate is 86.5% with mean intubation time of 40.4 s. The KVNC produced 24.8% success rate, with mean time to intubation of 53.2 s. The MG laryngoscope produced 85.0% success rate, with a mean time of intubation of 37.4 s.

**Conclusion:**

Military medical officers with 2–3 years of post-graduate training had a success rate of 71.4% success rate intubating a simulated difficult airway in a trauma setting using a DL. Success rates were improved with the use of KVC and the MG laryngoscope, but was worse with the KVNC.

## Background

Placement of a cuffed endotracheal tube (ETT) in the trachea remains the definitive airway management when resuscitating collapsed patients and treating severely injured trauma casualties [1, 2]. Direct Laryngoscopy (DL) is the primary method for tracheal intubation, but it becomes challenging when performed under emergency trauma conditions where casualties can have possible orofacial trauma or head and neck injuries. Repeated attempts and prolonged tracheal intubation can also result in significant morbidities, and this happens more frequently when tracheal intubation via DL is attempted by inexperienced operators or junior doctors [3–8]. The advent of video laryngoscopes (VL) has markedly improved visualization of the glottis via indirect laryngoscopy, and some studies suggest that VL may improve first-pass intubation success by non-experts [9, 10].

VLs play an important role in difficult airway algorithms [11, 12]. However, recent studies have called into question the utility of VL when intubating emergency and critical patients, and some studies suggest that VLs take longer, did not improve first-pass intubation rates, and may be associated with more complications compared to DL [13–17].

Our study aimed to evaluate the proficiency of tracheal intubation in simulated difficult airways using SimMan® 3G manikins by military medical doctors with 2 to 3 years of postgraduate clinical experience, and the extent to which VL can improve intubation success rates and timings.

## Methods

**Fig. 1:**
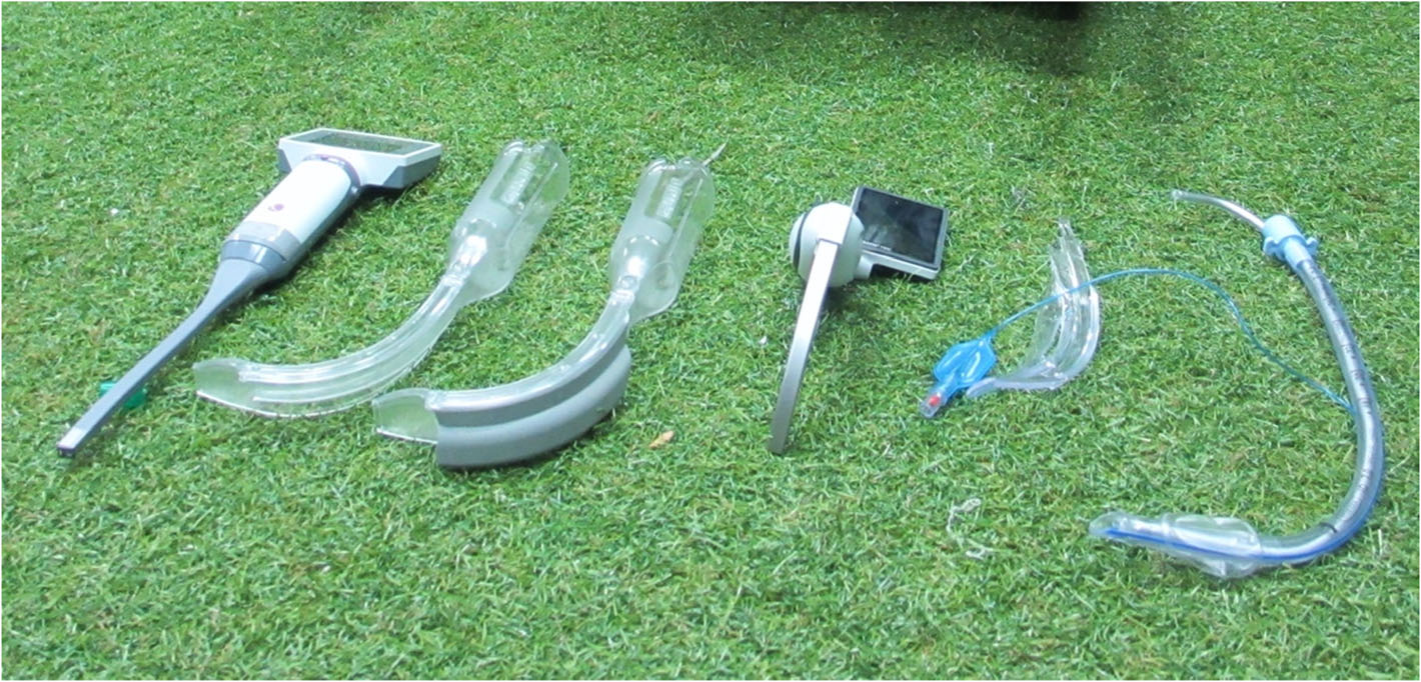
(From Left to Right) KingVision Video Laryngoscope with Non-Channeled and Channeled Blades, McGrath Video Laryngoscope, Endotracheal Tube with Stylet in-situ. (Photograph by Chin BZB, permission granted to use)

Our research was reviewed and granted approval by the institutional research committee of the Singapore Armed Forces. For the purpose of this study, the VLs selected had to be portable, battery-operated with an attached screen, relatively inexpensive, and come with disposable blades for single use appropriate for the trauma victim in an austere environment. Based on the above parameters, the research team selected the King Vision Laryngoscope and the McGrath Video Laryngoscope (MG), to be compared against the DL using a Macintosh size 3 blade in the management of simulated difficult airways (See Fig. 1). The King Vision Channeled Blade Laryngoscope (KVC) and the King Vision Non-Channeled Blade Laryngoscope (KVNC) were evaluated to investigate if channeled blade resulted in any differences in intubation success rates when used by junior medical officers with limited intubation experience.

For this study, we recruited 133 medical officer cadets of the Singapore Armed Forces (SAF) from 2016 to 2017. These doctors were 2 to 3 years post-graduate and would have undergone at least 3 months of anesthesia or emergency medicine department rotations. Written informed consent forms were obtained from all study participants prior to participation in the study.

**Fig. 2:**
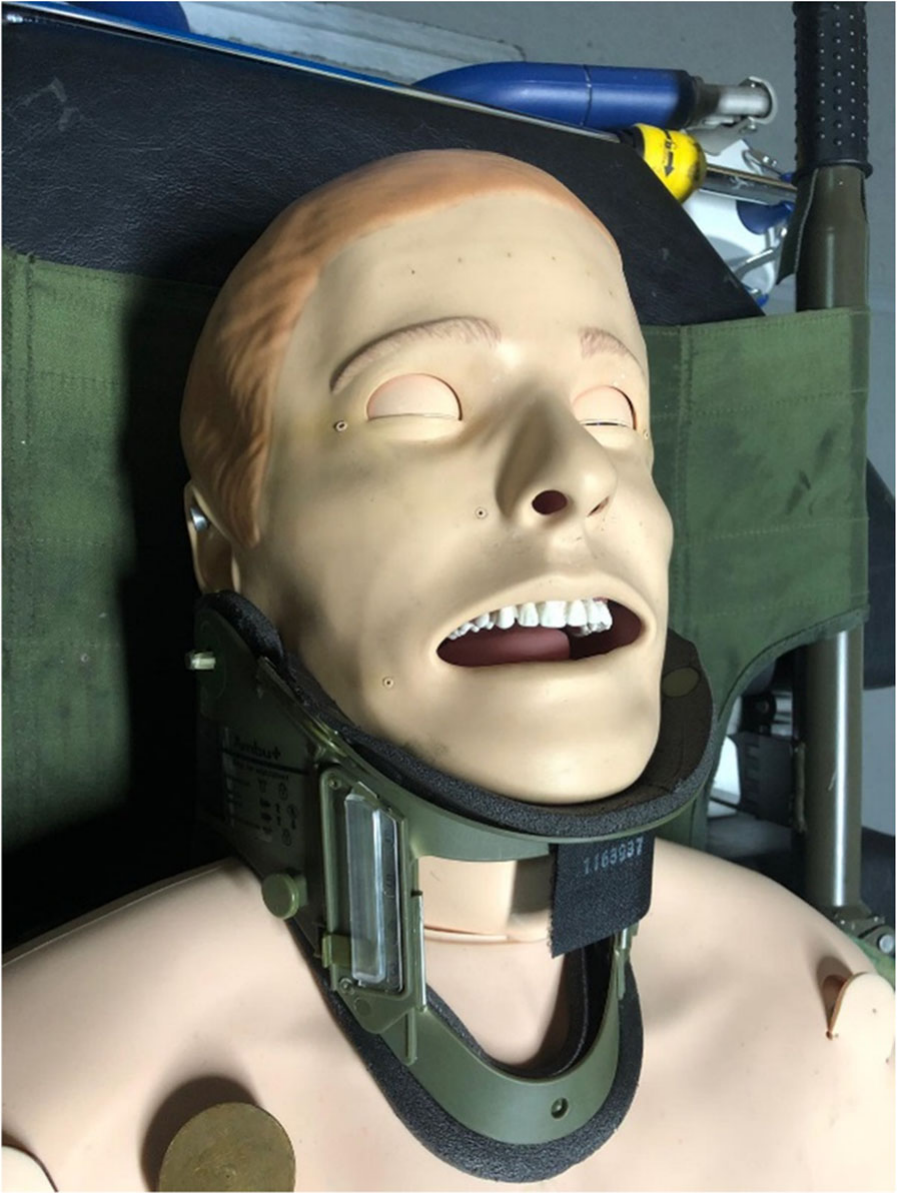
Manikin with simulated difficult airway (Tongue-swelling, cervical collar in-situ, and placed on a stretcher). (Photograph by Chin BZB, permission granted to use)

**Fig. 3:**
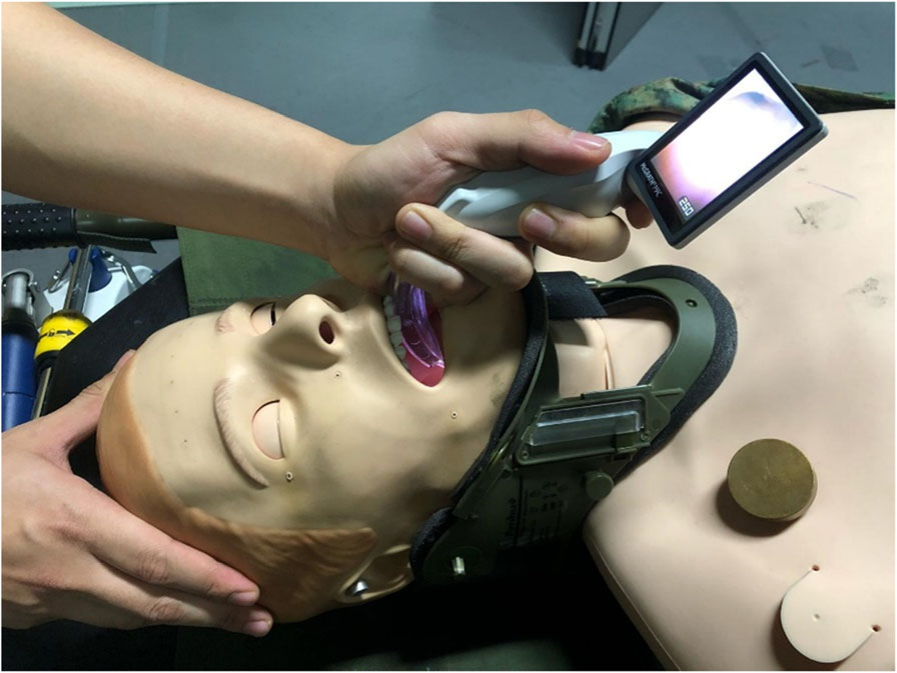
Attempting laryngoscopy using the McGrath on manikin with simulated difficult airway. (Photograph by Chin BZB, permission granted to use)

A standardized simulated difficult airway scenario was created for the participants by activating the tongue-swelling feature in the SimMan® 3G manikins with a standard cervical collar applied for cervical immobilization (See Figs. 2 and 3). To simulate trauma conditions, participants had to intubate the manikins placed on a stretcher lying on flat ground with a standard size #7 ETT.

All participants were shown a training video on the use of KVC, KVNC, and MG. They also had to undergo a familiarization session with the SimMan® 3G manikins where the authors demonstrated intubations on the manikins.

Each participant was given 1 attempt for direct laryngoscopy of the SimMan® 3G manikin placed on a stretcher on the ground for which the time to intubation (TTI) was recorded. TTI was defined as time from when the participant picks up the laryngoscope to when successful tracheal intubation was confirmed by chest rise with manual ventilation via the ETT. Each participant then progressed to use the KVC, KVNC and MG following a sequence based on a 3-period, 3-treatment crossover design (see Table 1) to minimize the learning effect of successive intubations on a similar manikin. This meant that Participant #1 was given sequence A, Participant #2 was given sequence B… and Participant #6 was given Sequence F. This cycle would restart again when we reached Participant #7, would be given Sequence A.

**Table 1:** 3-period, 3-treatment crossover design

Sequence	Period 1	Period 2	Period 3
A	MG	KVC	KVNC
B	MG	KVNC	KVC
C	KVC	KVNC	MG
D	KVC	MG	KVNC
E	KVNC	KVC	MG
F	KVNC	MG	KVC

In the event of oesophageal intubation, the participant was allowed to remove the ETT and re-attempt with no pause in the elapsed time, until either successful intubation was achieved or if 90 s had elapsed. A failed intubation was defined as failure to secure the ETT in the trachea within 90 s.

Data was collected and analyzed using Statistical Package for the Social Sciences (SPSS). Comparison of success rates was analyzed using Chi-squared tests. Analyses of continuous data (time to visualization and time to intubation) were done using paired t-test. A *p*-value of < 0.05 was considered significant.

## Results

A total of 133 doctors participated in this study. All participants had at least 3 months of emergency medicine or anesthesia rotation as part of their training requirement. The median number of previous successful intubations (using either DL or VL) based on recall amongst participants was 4.

Intubation success rates for DL was 71.4% (95 out of 133) with the mean time to intubation at 40.1 + 16.6 s. For the VLs, the intubation success rates were 86.5% (115 out of 133) for the KVC, with an average time taken of 40.4 + 20.2 s. For the KVNC, intubation success rate was 24.8% (33 out of 133) with an average time of 53.2 + 28.2 s. For the MG, intubation success rate was 85.0% (113 out of 133) with an average time of 37.4 + 19.3 s. (See Tables 2 and 3).

**Table 2:** Comparison of Intubation Success Rates between various VLs against the DL

	*Intubation success rates (%)*	*P-*value
*Direct Laryngoscope (DL) (n = 133)*	95/133 (71.4)	–
*Comparing success rates against Direct Laryngoscopy*	*Intubation success rates (%)*	*p-*value
*King Vision Channeled (KVC) (n = 133)*	115/133 (86.5)	0.0026
*King Vision Non-Channeled (KVNC) (n = 133)*	33/133 (24.8)	< 0.0001
*McGrath (MG) (n = 133)*	113/133 (85.0)	0.0073

**Table 3:** Comparison of intubation timings between various VLs against the DL

	*Time to Intubate(s)*^a^	*P-*value
*Direct Laryngoscope (DL) (n = 95)*	40.1 (+ 16.6)	–
*Comparing Time to Intubate against Direct Laryngoscopy*	*Time to Intubate(s)*^a^	*p-*value
*King Vision Channeled (KVC) (n = 115)*	40.4 (+ 20.2)	0.846
*King Vision Non-Channeled (KVNC) (n = 33)*	53.2 (+ 28.2)	0.014
*McGrath (MG) (n = 113)*	37.4 (+ 19.3)	0.192

^a^*Results expressed as mean (±standard deviation)*

Use of KVC and MG demonstrated higher intubation success rates for the difficult airway in a simulated trauma patient compared to DL. There was no significant difference in the time to intubation (TTI) between DL, KVC and MG when the intubation was successful. Comparing KVC with MG, we found that there was no significant difference in the intubation success rates and time to intubation (TTI).

## Discussion

All Singaporean male doctors are required to undergo at least 3 months of emergency medicine or anesthesia clinical rotations in order to improve their airway management skills prior to re-enlistment as military medical officers. Given that our scenario featured simulated difficult airways in trauma patients, we concluded that airway management proficiency of these junior doctors compared favorably to other large studies which reported success rates ranging between 71.2 to 85% for emergent intubations [18–20].

Chew et al. did a similar study on a cohort of military medical officers and found the intubation success rates were higher with channeled King Vision and McGrath as compared to the King Vision non-channeled laryngoscope [21]. This corroborated with our study findings.

In addition, our study showed that KVC and MG VLs were superior to DL in terms of intubation success rates, but did not significantly reduce the time to intubate in successful intubations. Several other studies also compared KVC/MG to DL [22–27]. Mehmet et al. demonstrated that MG produced a better view of the glottis, but the time to successful intubation was not significantly different from the DL [25]. Piepho et al. studied 30 paramedics using DL and MG on normal and difficult airway simulators, which demonstrated that the use of MG resulted in a better view of the glottis though success rates between MG and DL were similar [27]. Interestingly, Piepho’s study participants took longer time to intubation when they used MG, compared to DL [27]. Our team hypothesized that the superiority of VL over DL became more apparent with difficult airways. On the other hand, the familiarity of DL would be more advantageous when dealing with normal (easy) airways. For difficult airway scenarios, the first attempt at intubation tends to be the best attempt. This is because repeated attempts may result in laryngeal trauma and make intubation even more difficult. Hence, our study team recommends VL (KVC and MG) to be the first line laryngoscope for intubating anticipated difficult airways, especially in out-of-hospital settings.

The channelled conduit for ETT was designed to tackle the often-criticized problem of a ‘can see, but cannot intubate’ situation when trying to pass the ETT based on indirect visualisation of vocal cords when using VL [28, 29]. Our study echoed the findings of Akihisa et al., who demonstrated higher intubation success rate for KVC at 86.6%, compared to KVNC at 47.3% [29]. The same study also demonstrated an intubation success rate of 91.4% for DL, which proved to be better than for both KVC and KVNC [29].

Despite being a non-channeled VL, the MG compared very favourably compared to KVC, the channeled variant for the KingVision Laryngoscope. It also proved superior to the KVNC in terms of intubation success rate in our study. Most junior doctors in Singapore are familiar with the use of standard Macintosh laryngoscope, which is part of the standard equipment for securing the airway. We postulated that since the MG has a similar blade curvature and shape as the Macintosh DL, the MG VL was more intuitive and thus the success rates were higher. The KVNC utilised an acute-angle blade, which allowed for easy visualisation of the manikin vocal cords whilst the manikin was positioned on the floor. However, guiding the ETT through this acute angle was a tricky manouvre that most candidates failed to achieve, which is a similar problem seen in other studies on acute angled laryngoscopes [30, 31].

### Limitations

While the SimMan® 3G manikins used are high-fidelity advanced patient simulators, intubating a manikin remains different from intubating real patients. While the patient simulator can produce cervical immobilization and tongue edema, other difficult airway scenarios such as blood and secretions in the oral cavity, anatomic variations or mandibular injuries could not be simulated.

One possible bias in the study design was the ‘learning effect’ from successive intubations. With successive intubations, the study participant would be more familiar with the characteristics of the mannikin. This could possibly lead to higher success rates with subsequent intubations. Our study protocol required all participants to intubate with DL as their 1st attempt, before they were allowed to use video laryngoscopes. The ‘learning effect’ could possibly give VL an unintended advantage over DL.

Lastly, observation bias (aka Hawthorne Effect [32]) might lead to unexpectedly worse intubation success rates and timings for participants with performance anxiety, or improved intubation timings for participants who viewed this as a ‘time challenge’ and intubated much faster than they would have in real life situations.

## Conclusions

A plethora of airway trials have been done over the past decade comparing various VLs when used by either novice operators or experienced laryngoscopists (emergency medicine physicians, anesthesiologists, critical care medicine physicians). We hoped this study managed to shed more light on the proficiency of airway management of medical officers fronting the first-line response to airway emergencies in the military setting.

Though not the primary objective of the study, our results suggest that the blade curvature for non-channeled VLs contributed significantly to the success or failure of intubation by novice operators. The familiarity of blade curvature could also suggest that it would be easier to train junior doctors in Singapore to be adept with MG as compared to KVNC.

## Data Availability

Softcopy data has been anonymized and is kept with the authors. It is available upon request.

## Abbreviations

ETT: Endotracheal tube
DL: Direct laryngoscopy
VL: Video laryngoscopy
KVC: King-vision channelled blade laryngoscope
KVNC: King-vision non-channelled blade laryngoscope
MG: McGrath videolaryngoscope
TTI: Time to intubate
